# Sepsis in preterm infants causes alterations in mucosal gene expression and microbiota profiles compared to non-septic twins

**DOI:** 10.1038/srep25497

**Published:** 2016-05-16

**Authors:** María Cernada, Christine Bäuerl, Eva Serna, Maria Carmen Collado, Gaspar Pérez Martínez, Máximo Vento

**Affiliations:** 1 Health Research Institute (Instituto de Investigación Sanitaria) Hospital La Fe, Av. Fernando Abril Martorell 106; 46026 Valencia, Spain; 2 Division of Neonatology. University & Polytechnic Hospital La Fe, Avda. Fernando Abril Martorell 106; 46026 Valencia, Spain; 3 Institute of Agrochemistry and Food Technology, Spanish National Research Council (IATA-CSIC), Department of Biotechnology. Av. Agustin Escardino 7, 46980 Valencia, Spain; 4 Central Research Unit-INCLIVA, Faculty of Medicine, University of Valencia, Spain; 5 Spanish Maternal and Child Health and Development Network Retics Red SAMID, Health Research Institute Carlos III, Spanish Ministry of Economy and Competitiveness, Sinesio Delgado 4, 28029 Madrid, Spain

## Abstract

Sepsis is a life-threatening condition in preterm infants. Neonatal microbiota plays a pivotal role in the immune system maturation. Changes in gut microbiota have been associated to inflammatory disorders; however, a link with sepsis in the neonatal period has not yet been established. We aimed to analyze gut microbiota and mucosal gene expression using non-invasively obtained samples to provide with an integrative perspective of host-microbe interactions in neonatal sepsis. For this purpose, a prospective observational case-control study was conducted in septic preterm dizygotic twins and their non-septic twin controls. Fecal samples were used for both microbiota analysis and host genome-wide expression using exfoliated intestinal cells. Gene expression of exfoliated intestinal cells in septic preterm showed an induction of inflammatory and oxidative stress pathways in the gut and pro-oxidant profile that caused dysbiosis in the gut microbiota with predominance of *Enterobacteria* and reduction of *Bacteroides* and *Bifidobacterium* spp.in fecal samples, leading to a global reduction of beneficial anaerobic bacteria. Sepsis in preterm infants induced low-grade inflammation and oxidative stress in the gut mucosa, and also changes in the gut microbiota. This study highlights the role of inflammation and oxidative stress in neonatal sepsis on gut microbial profiles.

Early microbial gut colonization after birth strongly influences the maturation of the immune system^1^,^2^. The establishment of different bacterial populations will depend on maternal health status, antibiotic treatment, type of birth, but also from gestational age and the type of feeding^3^,^4^. Conditions causing alteration of the microbial balance in the neonatal period could expand their negative influence into later periods of life^5^.

Diseases of inflammatory nature have been directly associated to specific microbial signatures or with dysbiosis and conversely changes in the composition of the gut microbiota may have effects on the host and contribute to the development of diseases that involve inflammatory disorders^6^,^7^,^8^,^9^. Furthermore, the existence of a crosstalk between gut microbiota and the brain mediated by specific signaling pathways has been established^10^,^11^,^12^,^13^.

Sepsis is an extremely severe condition in the neonatal period. In preterm infants, the incidence ranges between 2% for vertical sepsis (mother-transmitted) and 20% for nosocomial (hospital-acquired) sepsis. Overall mortality is close to 18%^14^. Moreover, many survivors will suffer from neurodevelopmental and sensorial sequels^15^. Signs and symptoms of neonatal sepsis are extremely subtle rendering clinical diagnosis very difficult^16^,^17^,^18^. The etiologic diagnosis is based upon the isolation of a microorganism in the blood culture. *Coagulase-negative staphylococci* (CONS) followed by gram-negative bacteria are the most frequently identified pathogens^14^. However, blood culture frequently yields negative results due to low degree bacteremia, small inoculation volumes, and/or antibiotics supplied to the mother during labor^19^,^20^,^21^. Remarkably, sepsis affects gut homeostasis and consequently the gut microbiota. Moreover, following a septic process preterm infants exhibit a distorted microbiome with predominance of *Staphylococcus* species and reduced diversity with no specific enrichment of potential pathogens^22^,^23^.

Genome-wide expression profiles can discriminate septic from non-septic preterm infants in the neonatal period^24^. Gene expression analysis of exfoliated intestinal cells (EIC) and the transcriptional information obtained could disclose non-invasively relevant information about the biologic situation of the intestinal epithelial tissue^16^. However, studies of gene expression in EIC and microbiota in septic preterm infants have not been yet conducted.

The aim of the present study was to get an insight into the processes taking place in the gut of preterm infants during sepsis compared to their non-septic twins searching for possible relationships between changes in the gut microbiota and gene expression of EIC.

## Results

### Population

Five pairs of preterm twins (≈30 weeks’ gestation) were enrolled. Each pair included one twin who developed sepsis and a non-septic control. No other differences were observed between cases and controls (Table 1). Two of the neonates with sepsis had a positive blood culture test. The causal agent was identified as coagulase-negative *Staphylococcus* strain.

### Transcriptomic analysis of exfoliated epithelial cells

**Figure 1:**
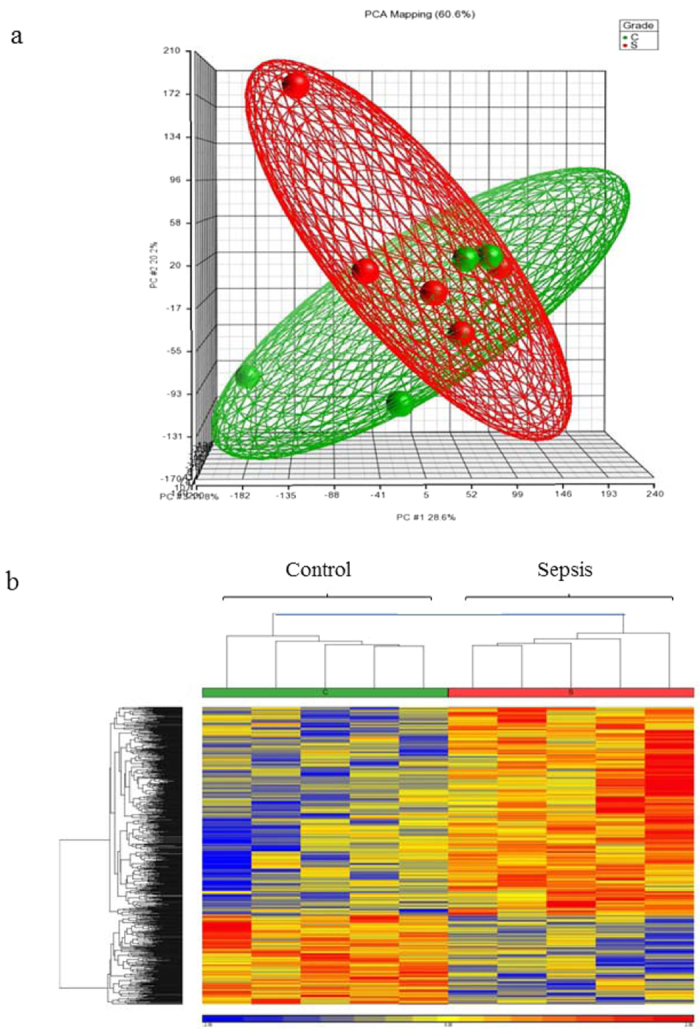
(Panel a) Principal component analysis (PCA) of the gene expression profiles of exfoliated intestinal cells of septic preterm twins (in red) and their non-septic controls (in green). The 3D plot shows the correlation of each sample with respect to the first three principal components. (Panel b) Represents the unsupervised hierarchical cluster analysis. Significantly regulated genes (p < 0.05) were used for 2-D hierarchical clustering of sepsis and control samples. Each row represents the expression of an individual probe set and each column of an individual sample, summarizing control samples with a green bar and sepsis samples with a red bar. Upregulated genes are represented in red and downregulated genes in blue.

Total RNA from the fecal samples of the infants was hybridized with whole human genome microarrays (28,000 annotated genes). Three-dimensional unsupervised principal component analysis (PCA) showed two clustered groups that included the septic and non-septic control samples (Fig. 1a). Further analysis yielded 819 differentially expressed probe sets (ANOVA, p-value < 0.05) between septic and non-septic infants (Tables S1 and S2). Only 510 annotated genes were considered from which 343 genes were up-regulated and 167 were down-regulated. The unsupervised hierarchical clustering showed sensitive differences in gene expression between septic infants and non-septic controls (Fig. 1b). Table 2A shows the most up and down-regulated genes in both groups. Proteins encoded in the list reflect certain degree of inflammation and infiltration of immune cells in the gut mucosa (fecal up-regulation of CD40LG during sepsis). These data are in agreement with gene expression in peripheral blood cells of preterm infants with sepsis^24^ (Table 2B).

### Functional annotations, upstream regulators and signaling pathways

According to Pathway Studio (PS) most of the induced gene expression changes were related to regulation of transcription (Table 3A). The most significant were *DNA-dependent regulation of transcription* (P = 2.53E-08) representing typical interaction modules with DNA and RNA (Table S3), and HDAC2 encoding for histone acetylase 2^25^. The annotation term *metabolic process* entailed a large number of genes encoding different enzymes involved in ATB-binding (Table S3) and oxidative stress ((glutathione peroxidase 1 (GPX1); glutathione S-transferase mu2 (GSTM2))^26^,^27^.

**Figure 2:**
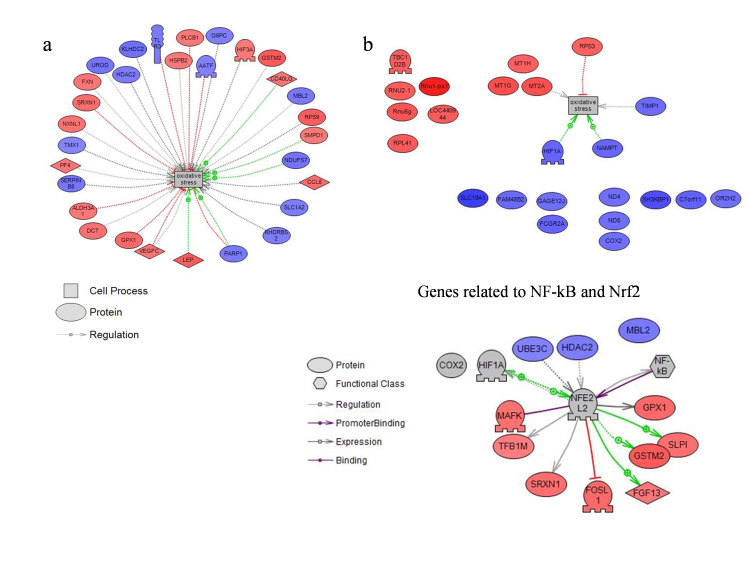
Pathway Studio Analysis Protein-protein interaction network. (Panel a) Genes correlated with Oxidative Stress Pathway. (Panel b) Genes linked with the NF-кB canonical pathway.

Further, protein-protein networks and signaling pathways could be inferred from expression data. Annotated genes showing significant up- and down-regulation with greater fold changes (FC > 2 and FC < −2) were selected for the PS analysis. They revealed interaction pathway related to Oxidative Stress (Fig. 2, Panel a) and its connection to the canonical NF-κB inflammatory pathway (Fig. 2, Panel b). Upstream regulator analysis (Table 3B) identified FOXN4 and SP1 as most significant transcription regulators.

**Figure 3:**
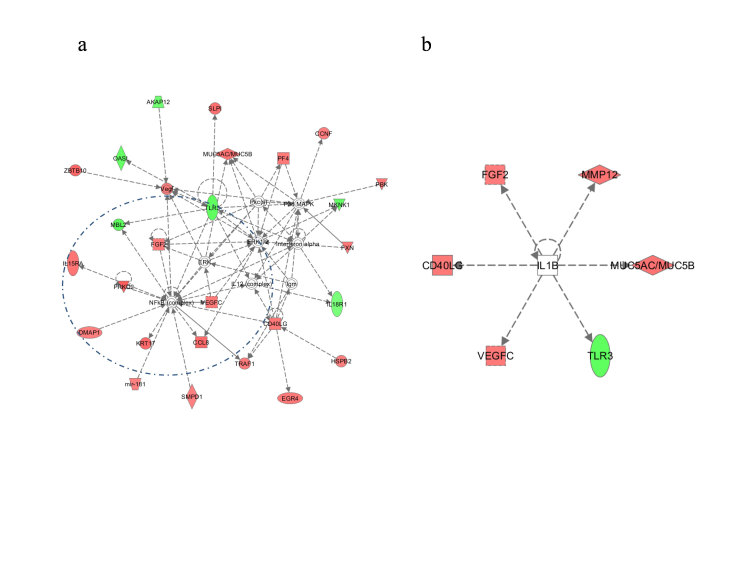
IPA analysis: (Panel a) Top Network Cell Death and Survival, Inflammatory Response, Cancer. (Panel b) Upstream regulation: evidence of the activation of IL1B pathway. IL1B predicted to be activated (z-score 2.368) p-value = 1.84E-01: 6 out of 6 genes have expression direction consistent with activation of IL1B.

Ingenuity Pathway Analysis (IPA) provided a functional analysis with gene Networks conceptually complementary to the biological processes and master regulators described above (Table 3C). The most significant interaction network related a collection of genes to the molecular functions *Cell Death and Survival, Cancer and Inflammatory Response* (Fig. 3, Panel a). In agreement with PS, using the upstream regulator analysis tool of the IPA software a contribution to regulatory role could be again attributed to IL1-β, which predicts IL1-β to be activated as transcriptional regulator (z-score 2.368), although, due to the low number of activated genes in the pathway there was a low significance score (P = 0.18) (Fig. 3, Panel b).

### Microbial diversity and composition determined by molecular methods

**Figure 4:**
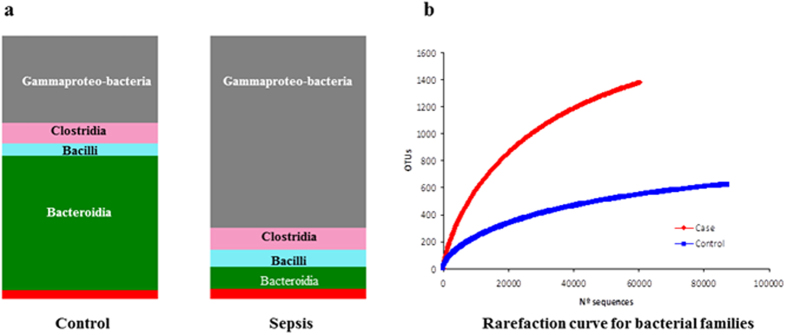
Microbiota composition obtained by pyrosequencing. (Panel a) Relative abundance of bacterial distribution at class level in the Sepsis and Control groups. (Panel b) Rarefaction curves at level of 90% for families. The graphs shows rarefaction curve relating the sequencing effect compared with an estimate of the number of bacterial families, as inferred by the number of OTUs.

A primary analysis of the main bacterial groups performed by qPCR of 16S rDNA showed differences between septic and control twins (Fig. 4a). Healthy controls exhibited higher amounts and prevalence of *Bifidobacterium* species (P = 0.050 and P = 0.055, respectively) and lower levels of *Enterobacteriaceae* (P = 0.093). Although *Enterobacteriaceae, Enterococcus* and *Staphylococcus* groups were common in the septic group, prevalence had low significance scores (P > 0.05).

By pyrosequencing, we obtained an average of 10.265,5 (SD ± 4957.04) sequences per infant sample. The average length per sequence was 425 nucleotides (range 300–455). Table S4 shows the predominant phyla present in all samples. Thus while *Lactobacillales* and *Enterobacteriales* showed higher frequency in septic patients *Bacillales* were lower. Pseudomonadales and Vibrionales were found only in the septic group.

At family level, *Enterobacteriaceae* proportions were higher (72.2% vs. 30.4%, respectively) and *Bacteroides* were lower in sepsis (18.6% vs. 21.7%, p-value = 0.192) than in controls. A greater proportion of *Enterococcaceae* was found in sepsis (5.5%) than in the control group (<0.1%). We found a significantly higher percentage of *Staphylococcaceae* sequences in control (4.2%) than in sepsis cases (<1%); however, proportions were low.

We observed that the septic group showed higher bacterial diversity than the control group although differences were not significant (Fig. 4b). Moreover, taxons of twin neonates suffering sepsis included predominantly aerobic or aerotolerant species with a decrease of strict anaerobes.

### Associations between microbes and neonatal intestinal gene expression

**Figure 5:**
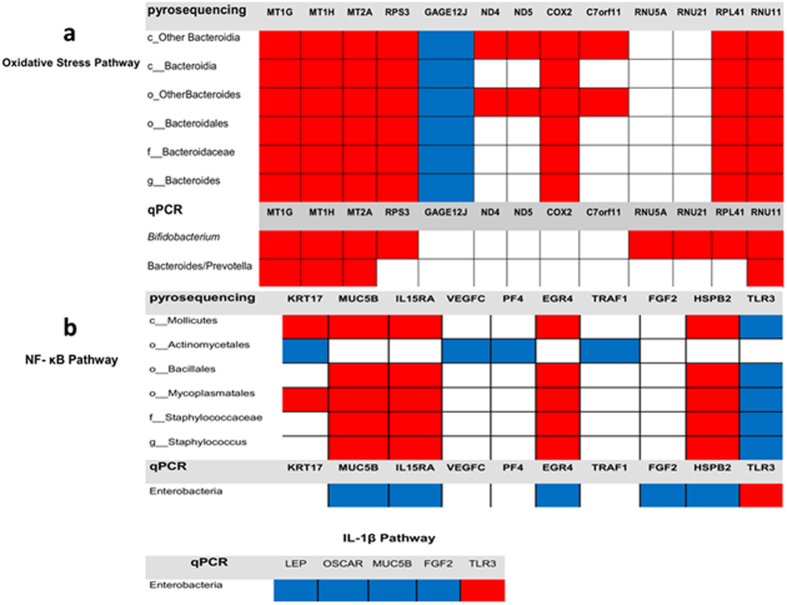
Depicts the colored representation of the correlation between bacterial groups (reads of pyrosequencing of numbers estimated by qPCR) and expression (signal intensity) of the differentially expressed genes in the Oxidative Stress pathway (panel a) and in the NF-κB pathway and IL-1β pathways (panel b). Red represents significant inverse correlation and blue cells’ significant direct correlation. Genes with white cells gave no significant correlation with the corresponding bacterial taxon and genes not shown had no significant correlation with any bacterial taxa.

First we analyzed both the sets of genes involved in inflammation and oxidative stress (except SLC19A1 and OR2H2) to establish a correlation between these and the most prominent bacterial clades in septic patients (Fig. 2). Pearson’s correlation of the expression of oxidative stress-related genes with significant clades revealed a significant inverse correlation between the order Bacteroidales, family *Bacteroidaceae* and genus *Bacteroides* and 8 of 21 genes in the oxidative stress pathway (11 of 21 genes if we include “Other Bacteroidales”) (Fig. 5a, Table S5A). Results were confirmed with data obtained by qPCR, as a significant inverse correlation with the Bacteroides/Prevotella group was confirmed with 4 of 21 genes. Interestingly, qPCR data also indicated a significant inverse correlation between the calculated reads of the genus *Bifidobacterium* and the expression of oxidative stress genes (8 of 21) (Fig. 5b, Table S5A). Both genera *Bifidobacterium* and *Bacteroides* include strict anaerobic species. Other bacterial groups decreased significantly when signal transduction pathways controlled by the master regulator NF-κB were active such as the order Mycoplasmatales and the genus *Staphylococcus* with significant correlation with 6 and 5 genes respectively out of 15 (Fig. 5b, Table S5B). Enterobacteria counts obtained by qPCR directly correlated with a moderate activation of the NF-κB and IL-1β pathways (6 and 5 of 15 genes, respectively).

## Discussion

The analysis of intestinal cells and gut microbiota in the present study offers an integrative perspective of the events occurring in the gut of preterm infants during sepsis considering that inter-individual differences were minimized by the selection of dizygotic twin pairs with and without sepsis. Gene expression differences between septic and non-septic infants were moderate, between 2 and −2, because EIC integrate pools of various types of cells –epithelial cells, lymphocytes- at different physiological conditions, including anoikis. Despite this variability, our data demonstrated that EIC from septic patients significantly expressed more genes bound to inflammatory pathways and oxidative stress than non-septic controls. Of note, none of the analysis performed showed significant differences between culture positive samples and those with clinical diagnostic.

Some pro-inflammatory genes were up-regulated in EIC revealing the implication of inflammatory regulators; however, these genes do not participate in canonical responses to gut bacteria as pathogen-associated molecular patterns. The infiltration of lymphocytes in feces may partially explain finding of just some differentially expressed genes in exfoliated cells similar to those reported in blood of septic preterm infants^24^ (Table 2) as expected in the case of inflammatory organ crosstalk. Genes showing a similar trend in EIC and peripheral blood – like OSCAR, ARID5A SMYD2 and CD3e- may probably reveal the presence of circulating immune cells in feces. OSCAR encodes a collagen receptor that in monocytes and neutrophils plays a role in the release of pro-inflammatory cytokines and oxidative stress mediators^28^. ARID5A contributes to the stabilization of the mRNA encoding the pro-inflammatory cytokine IL-6^29^,^30^. SMYD2 encodes a mono-methyl-transferase that methylates acceptor lysine residues on histones^31^,^32^ and other proteins like tumor suppressor proteins p53 and pRb relevant to tumor progression and/or undetermined epigenetic events^33^,^34^,^35^. Finally, CD3E down-regulation may indicate that antigen recognition may be uncoupled during sepsis in CD4^+^ T lymphocytes and T-cells^36^.

Functional annotation analysis offered intriguing clues on possible connections to epigenetic modifications. In septic infants we found activated biological processes like *gene transcription* that involved down-regulation of HDAC2 a histone acetylase^34^,^35^ and PARP1^37^ but also up-regulation of SMYD2 that methylates histones^31^,^32^, and DNA methyltransferase associated protein (DMAP1). Histone deacetylases can modulate gene expression through endogenous factors, but also through dietary components, synthetic inhibitors, and bacteria-derived signals^38^,^39^, thereby underlining the mutualistic relationship that exists between gut microbiota and host.

**Figure 6:**
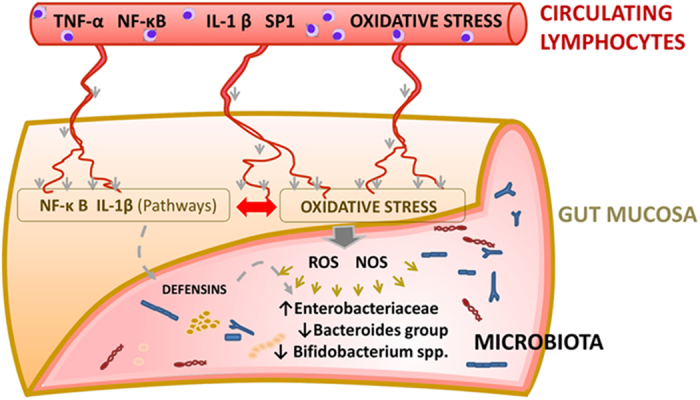
Model explaining changes in gene expression and the gut. Circulating lymphocytes expressed high levels of inflammatory markers (TNF-α, NF-κB, IL-1β and SP1). High amounts of innate immune mediators secreted to the bloodstream would be reaching the mucosae of the small and large intestine of VLBW infants with sepsis. This would activate master regulators TNF-α and IL-1β, inducing the expression of pro-inflammatory signaling and innate immune defense systems, such as oxidative stress pathway in the gut mucosal cells. The secretion of ROS and NOS would then correlate with bacterial profiles richer in *Enterobacteriaceae* and with the lower presence of *Bifidobacteriaceae* and *Bacteroides*, due to the lack in the latter of the enzymatic armor to survive in the presence of ROS, RNS and derived toxic compounds.

The finding of active oxidative stress pathways in exfoliated cells led us to reanalyze data obtained from peripheral blood cells of VLBW infants, and we found significant values for the oxidative stress pathway (p = 0.0015) that involved the genes BCL2, LCN2, MMP9, EPAS1, BTG1, TOR1 and VNN1^24^. The activation of the oxidative pathways in gut cells could be initiated by the circulating inflammatory mediators or by reactive oxygen (ROS) or nitrogen (RNS) species secreted by blood lymphocytes^40^. Metabolomic analysis has shown that sepsis in the neonatal period is associated to an increase in oxidative stress biomarkers^41^. ROS and RNS intermediates are powerful antimicrobials and major components of the innate defense frontline; however, beyond a threshold they have deleterious effects. Remarkably, oxidative stress has been related to changes in the microbial patterns in the gut^42^,^43^. Thus, we have observed a decrease of strict anaerobes (*Bacteroides, Bifidobacterium*) in the septic group, followed by the proliferation of aerobes (*Enterobacteriaceae*). The genera belonging to this family include species equipped with specialized enzymes such as tetrathionate reductase^44^ and other versatile cytochrome related enzymes that allow the use of ROS-oxidized molecules as electron acceptors^45^. Other bacterial groups found in the sepsis samples can reduce nitrate and nitrite to NO, or have catalase or pseudo-catalase enzymes^46^. Altogether these properties would grant them competitive advantage. A general model can be inferred in septic preterm infants that would allow understanding of the likely relationship between blood lymphocyte inflammatory signals during sepsis, with expression of inflammatory and oxidative stress genes, as well as changes in the gut microbiota (Fig. 6). In blood cells, master regulators induce canonical inflammatory pathways^24^ and oxidative stress elicited by bacterial septic invasion, leading to the secretion of abundant pro-inflammatory cytokines. Through inflammatory organ crosstalk that led to low but significant expression of inflammatory networks and oxidative stress genes in the gut mucosa –exfoliated epithelial cells- that drives the secretion of ROS and NOS to the lumen, hence affecting the viability of sensitive bacterial species, like *Bacteroides* and *Bifidobacterium* species.

To summarize, this pilot study shows that sepsis in preterm infants induces inflammation in the gut mucosa and changes in gut microbiota extending inflammation-associated damage to sensitive organs. Our results also support the relevance of oxidative stress in sepsis, which may cause the reduction of *Bifidobacterium* spp. and other beneficial anaerobes in the gut lumen. These data will open new possibilities to reduce the neonatal sepsis using the knowledge and potential role of human breast milk acting as a “gut protector” and antioxidant^47^.

## Methods

### Patients and Design

We conducted a prospective, observational, cohort study in the NICU of the University and Polytechnic Hospital La Fe from April 2011 to September 2012. Eligible patients were twin pairs with a birth weight ≤ 1.500 g. When one of the twins exhibited clinical signs of sepsis, the other was simultaneously recruited as control if lacking signs of sepsis to homogenize the genetic background. Sepsis was considered clinical signs such as the presence of 3 or more of the following: (i) temperature instability (rectal temp. >38 °C or <36 °C); (ii) respiratory symptoms (respiratory distress, apnoea or cyanosis);

(iii) cardiovascular symptoms: hypotension (blood pressure <5th percentile for age), tachycardia (HR > 0180/min), bradycardia (HR < 100/min) or poor perfusion;

(iv) neurological symptoms: clinical or electrical seizures, hypotonia or lethargy; and (v) gastrointestinal symptoms: vomiting, poor feeding or feeding intolerance and/or

abdominal distension, without identification of a bacterial pathogen from a sterile site. Exclusion criteria were: maternal immunodeficiency; Apgar 0 at 1 min; chromosomal abnormalities or major gastro-intestinal malformation; congenital infection; concomitant diagnosis of necrotizing enterocolitis.

The clinical interventions included in the study protocol were designed according to the Clinical Guidelines approved by the Spanish Neonatal Society (SENeo) and the Ethical Guidelines of the *Comité de Ética e Investigación Médica* (CEIM) of the Health Research Institute La Fe. The study protocol was approved by the CEIM. The inform consent was signed by parents of all the enrolled patients.

### Diagnosis of sepsis

A standard blood culture was performed (BacT/Alert^®^ PF; Biomérieux^®^, Durham, NC; USA) only in patients with suspected sepsis. Microbiologic positive diagnosis was considered when a microorganism was isolated from blood culture together with clinical signs and risk factors^48^. Two positive blood cultures were required to diagnose CONS sepsis. Cultures obtained from other sterile sites were not considered for diagnosis of sepsis.

### Samples

Stool samples from both twins were collected immediately after sepsis was suspected. Samples were frozen and stored at −80 °C for later analysis.

### RNA extraction and cDNA microarray analysis

Total RNA of EIC was isolated from fecal samples using the Trizol reagent (TRIzol, Invitrogen.), followed by an polyA+ RNA enrichment step in order to eliminate contaminating DNA and bacterial RNA using the mRNA-ONLY™ Eukaryotic mRNA Isolation Kit (Epicentre) according to the manufacturers´ instructions. RNA integrity was assessed using the Agilent 2100 Bioanalyzer (Agilent, Palo Alto, CA, USA). Hybridization was performed if the RNA integrity number was ≥7.

Microarray experiments were performed at the Central Research Unit (University of Valencia) using GeneChip Human Gene 1.0 ST Array (Affymetrix®, Santa Clara, CA, USA) following the manufacturer’s protocol. The files obtained (.CEL) were used to analyze significant changes in gene expression profiles using Partek Genomic Suite 6.6 (Partek Inc., St. Louis, MO, USA). Data were normalized using robust multiple-array average (RMA) algorithm. Principal Component Analysis (PCA) determined global transcriptome differences between samples. Next, 1-way ANOVA (p-value < 0.05) was applied to identify significantly different expressed genes between bacterial sepsis and control groups, and were ordered according to their expression levels in an unsupervised hierarchical clustering. To obtain insights about the pathways, biological processes and molecular functions differentially expressed in the sepsis group were investigated through functional annotation analysis with specialized computer packages Pathway Studio 9 (Ariadne Genomics® software, Elsevier® Inc, Rockville, MD, USA) and Ingenuity Pathway Analysis (Qiagen, Redwood City, CA, USA) that are based on different algorithms and concept definitions and would offer a wider perspective^49^.

### DNA extraction and microbial composition by quantitative PCR (qPCR)

DNA was extracted from 200–300 mg fecal samples using a modified Qiagen stool DNA extraction kit (QIAgen, Hilden, Germany), and qPCR amplification and detection were performed in a LightCycler® 480 Real-Time PCR System (Roche) as previously described^50^. The bacterial concentration in each sample was calculated by comparing the Ct values obtained from standard curves. These were created using serial 10-fold dilution of pure culture-specific DNA fragments corresponding to 10 to 10^9^ number of gene copies/ml. For statistical analysis, SPSS 15.0 (SPSS Inc, Chicago, IL) software was used. Due to non-normal distribution, microbial data are expressed as medians with interquartile ranges (IQR). The Kruskall-Wallis test was used to compare data of more than two groups of babies, while Mann-Whitney U test was used for comparing data of two groups. The χ-square test was used to establish differences in the prevalence of bacteria between the groups. A p < 0.050 was considered statistically significant. The possible correlation between variables was studied by applying the Pearson correlation coefficient and significance was established at 0.5%.

### Microbial diversity and composition estimated by pyrosequencing total 16S rRNA genes in feces

A barcoded primer set based on universal primers 27F and 533R was used to amplify 500 bps of the 16S rRNA genes encompassing the V1-V3 region. PCR was carried out using a high-fidelity KAPA-HiFi polymerase (Kappa Biosystems, US) with an annealing temperature of 52 °C and 30 cycles. Amplicons were checked and measured using the Agilent High Sensitivity DNA assay in Agilent 2100 Expert. Purified PCR products were pyrosequenced from the forward primer end only using a GS-Junior sequencer with Titanium chemistry (Roche) at the Scientific Platform unit SCSIE (University of Valencia, Spain).

From the resulting raw data set, low quality sequences or sequences with a length less than 150 nucleotides were discarded and also, chimeric sequences were removed. The QIIME pipeline (version 1.8.0 and green genes data base 13_5) was used for identifying representative sequences for each operational taxonomic unit (OTU) generated from complete linkage clustering with a 97% similarity. Alpha diversity indices were determined from rarefied tables using the Shannon-Wiener index for diversity and the Chao1 index for species richness; Observed Species (number of unique OTUs) and Phylogenetic Distance (PD_whole) were also determined. An OTU was a cluster of 16S rRNA sequences that were >95% identical, a conservative estimate for the boundary between species, established at 97% (species) and 95% (genus) for full-length 16S gene sequences. A beta diversity distance matrix was computed from the previously constructed OTU table using UniFrac analysis. Unweighted (presence/absence matrix) and weighted (presence/absence/abundance matrix) UniFrac distances were used to construct two- and three-dimensional Principal Coordinates Analysis (PCoA) plots. Biplots were generated as part of the beta diversity analysis in QIIME, using genus level OTU tables showing principle coordinate sample clustering alongside weighted taxonomic group data. Data on assigned sequences at genus level shared between samples were used to generate a Venn diagram^51^,^52^,^53^,^54^,^55^,^56^,^57^,^58^,^59^,^60^.

## Additional Information

**How to cite this article**: Cernada, M. *et al*. Sepsis in preterm infants causes alterations in mucosal gene expression and microbiota profiles compared to non-septic twins. *Sci. Rep.* **6**, 25497; doi: 10.1038/srep25497 (2016).

## Supplementary Material

Supplementary Information

## Figures and Tables

**Table 1:** Perinatal characteristics of preterm twin infants with (cases) and without (controls) neonatal sepsis.

Variables	Sepsis (N = 5)	Non Septicb Controls (N = 5)	P- value
Gestational age (weeks) (mean ± SD)	30 ± 1	30 ± 1	1.00^$^
Antenatal steroids full course	4 (80%)	4 (80%)	1.00^&^
Type of delivery (%)			1.00^&^
Vaginal	1 (20%)	1 (20%)	
C-Section	4 (80%)	4 (80%)	
Gender (%)			
Male	1(20%)	3 (60%)	0.524^&^
Birth weight (g) (mean ± SD)	1346 ± 266	1484 ± 97	0.308^$^
Race (%)			1.00^&^
Caucasian	4 (80%)	4 (80%)	
Black	1 (20%)	1 (20%)	
Breastfeeding	5 (100%)	5 (100%)	1.00^&^
Apgar Score 1 min (median; 5–95% CI)	8 (7–8)	7 (5–8)	0.583^$^
Apgar Score 5 min (median, 5–95% CI)	10 (9–10)	8 (8–9)	0.066^$^
Age at sample collection (days) (median, 5–95% CI)	11.(4–19)	17 (4–29)	0.443^$^
Weight at sample collection (g) (mean ± SD)	1308 ± 311	1560 ± 373	0.304^$^
FiO_2_ at sample collection (mean ± SD)	0.22 ± 0.018	0.21 ± 0.009	0.667^$^

^$^t-Student test, ^&^Fisher’s test two-sided.

**Table 2:** Up- and down-regulated genes in preterm infants with sepsis ordered by expression value (Panel A) and Comparison of gene expression (fold change) between data obtained from feces in the present study in preterm infants with sepsis and those from blood cells also in septic preterm newborn described in a previous study (Panel B).

Panel A: Up-and-Down regulated genes		p-value	Fold Change	
UPREGULATED GENES				
TBC1D2B	similar to TBC1 domain family, member 2B	0.0404223	2.105	
C3orf79	hypothetical protein LOC152118	0.0435912	1.896	
ZNF487P	zinc finger protein 487, pseudogene	0.0325316	1.789	
PSG4	pregnancy specific beta-1-glycoprotein 4	0.049411	1.649	
GSTM2	glutathione S-transferase mu 2(muscle)	0.0199524	1.618	
LEP	leptin	0.0307164	1.582	
CLK2	CDC-like kinase 2	0.0464448	1.578	
AQP7	aquaporin 7	0.0271608	1.559	
RPS9	ribosomal protein S9	0.0403301	1.554	
*RNU11*	*RNA, U11 small nuclear*	0.0348921	1.513	
Greatest significance level				
CD40LG	CD40 ligand	6.6 × 10^5^	1.209	
FAM58A	family with sequence similarity 58, member A	2.4 × 10^4^	1.463	
CPEB1	cytoplasmic polyadenylation element binding protein	9.8 × 10^4^	1.208	
C7orf70	family with sequence similarity 220, member A	0.0012	1.428	
FARS2	Phenyl-alanyl-tRNA synthetase 2, mitochondrial	0.0016	1.193	
DOWNREGULATED GENES		p-value	expression value	
ZNRD1	zinc ribbon domain containing 1	0.0036632	−2.012	
TRAPPC3	trafficking protein particle complex 3	0.0152393	−1.813	
SLC25A44	solute carrier family 25, member 44	0.0426276	−1.782	
LCE2C	late cornified envelope 2C	0.032163	−1.676	
LCN1	lipocalin 1	0.0386323	−1.660	
AMZ2P1	archaelysin family metallopeptidase 2 pseudogene 1	0.0462527	−1.649	
SYNJ2BP	synaptojanin 2 binding protein	0.0189059	−1.582	
C1QTNF9B	C1q and tumor necrosis factor related protein 9B	0.0223451	−1.574	
SLC45A4	solute carrier family 45, member 4	0.0449936	−1.538	
LARP4	La ribonucleoprotein domain family, member 4	0.0393834	0.670	
Greatest significance level				
PSKH2	protein serine kinase H2	0.0010	−1.257	
LONP2	lon peptidase 2, peroxisomal	0.0016	−1.297	
TSTD2	thiosulfate sulfurtransferase (rhodanese)-like domain containing 2	0.0019	−1.305	
ZNF514	zinc finger protein 514	0.0024	−1.120	
OAZ2	ornithine decarboxylase antizyme 2	0.0032	−1.211	
**Panel B)**				
	**Feces**	**Blood (Ref.** 24)		
	**p-value**	**Fold change**	**p-value**	**Fold change**
C7orf70	0.00119	1.43	0.000796	−1.25
OSCAR	0.02477	1.29	0.000388	1.39
ARID5A	0.03141	1.29	0.000002	1.47
CD40LG	0.00007	1.21	0.000069	−2.20
IL18R1	0.01898	−1.07	0.000004	3.15
SMYD2	0.02760	−1.12	0.000432	−1.60
HP	0.04761	−1.20	0.000082	4.17
TBC1D8	0.00796	−1.27	0.00013	2.17
CD3E	0.032	−1.27	0.000783	−1.96

(see ref. 24).

**Table 3:** Functional analysis was performed with two pathway analysis platforms that render complementary information: Pathway Studio and Ingenuity Pathway Analysis.

(A) Functional annotation analysis (Pathway Studio): Top ten modulates biological processes		
Biological process	# Genes	Significance (p-value)
Regulation of transcription, DNA-dependent	64	2.53E-08
Metabolic process	57	2.67E-08
Transcription, DNA-dependent	53	1.09E-07
Transport	46	8.44E-08
Response to drug	20	4.46E-07
Transmembrane transport	20	8.72E-04
Multicellular organismal development	20	2.68E-02
Positive regulation of cell proliferation	17	7.50E-06
Negative regulation of transcription, DNA-dependent	16	3.32E-05
Negative regulation of transcription from RNA polymerase II promoter	15	4.90E-04
**(B) Upstream regulator analysis (Pathway Studio): Ten most significant master regulators**		
**Master regulator**	# Genes	**p-value**
FOXN4	4	5.19E-05
SP1	36	1.15E-03
NR1H2	5	1.42E-03
Endothelin	7	1.77E-03
IL1 family	19	3.56E-03
FOXA3	3	4.49E-03
MIR1-1	9	5.08E-03
SP3	14	5.20E-03
PBX1	4	5.52E-03
ferritin	3	6.71E-03
**(C) Ingenuity Pathway Analysis**		
**ID**	**Associated Network Functions with highest scores**	**Score**
1	Cell Death and Survival, Inflammatory Response, Cancer	36
2	Cell Morphology, Cell Death and Survival, Cellular Movement	30
3	Cell Death and Survival, Tumor Morphology, Cellular Development	21
4	Dermatological Diseases and Conditions, Hematological System Development and function, Organismal Functions	16
5	Cell Death and Survival, Cancer, Cell Cycle	14
