# Supplementary material for: Sepsis in preterm infants causes alterations in mucosal gene expression and microbiota profiles compared to non-septic twins
Source: Sci Rep. 2016 May 16;6:25497. doi: 10.1038/srep25497 (PMC4867619; doi:10.1038/srep25497)
Supplement: Supplementary Information [file srep25497-s1.pdf]

SUPPLEMENTARY MATERIAL

TITLE

Sepsis in preterm infants causes alterations in mucosal gene expression and microbiota profiles compared to non-septic twins.

AUTHORS

María Cernada [a,b*], Christine Bäuerl [c], Eva Serna[d], Maria Carmen Collado[c], Gaspar Pérez Martínez [c*]; Máximo Vento [a,b,e**] (* These authors contributed equally to this work).

[a] Health Research Institute (Instituto de Investigación Sanitaria) Hospital La Fe, Av. Fernando Abril Martorell 106; 46026 Valencia, Spain.

[b] Division of Neonatology. University & Polytechnic Hospital La Fe, Avda. Fernando Abril Martorell 106; 46026 Valencia, Spain.

[c] Institute of Agrochemistry and Food Technology, Spanish National Research Council (IATA-CSIC), Department of Biotechnology. Av. Agustin Escardino 7, 46980 Valencia, Spain.

[d] Central Research Unit-INCLIVA, Faculty of Medicine, University of Valencia, Spain. [e] Spanish Maternal and Child Health and Development Network Retics Red SAMID, Health Research Institute Carlos III, Spanish Ministry of Economy and Competitiveness, Sinesio Delgado 4, 28029 Madrid, Spain.

Table S1.- Functional annotation analysis performed with Pathway Studio, where Biological Processes are related to the respective list of genes**.** The table shows 50 Biological Processes with highest significance (p-value) and overlapping.

Name	Type	Total Entities	Expanded # of Entities	Overlap	Percent Overlap	Overlapping Entities	p-value	Data Source	#
**regulation of transcription, DNA-dependent**	Group	2872	2872	64	2	ZNF16,ZNF184,ATP1B4,ZNF576,ZNF514,SCRT2,ZNF502,ZNF320,ZNF549,ZNF619,ZNF660,FOXI2,ZNF880,ZNF487P,FGF2,PARP1,FOSL1,KLF9,WDR5,PROX1,HDAC2,LRP6,HIF3A,GCM1,MTA3,TBX2,NFIC,ZBTB7A,CENPB,ZNF350,TMPO,RLIM,SMYD2,PTF1A,ZNRD1,ZBTB10,HSF2,MBD1,FOXA3,TLX2,TFB1M,BRF2,CBFA2T2,HTATSF1,EGR4,RFXANK,KHDRBS2,DMAP1,BCORL1,SKOR1,VGLL4,DRAP1,BHLHE22,JMJD1C,FIZ1,ZNF395,ARID5A,MTERFD3,ZNF296,MAFIP,MAFK,ZNF140,NCOA5,ZNF193	2.53E-08	biological _process	1
**metabolic process**	Group	2421	2421	57	2	DNAH17,PARP6,EPHX3,GALNTL4,PIK3CG,CES1,CDK6,ABCG5,LCLAT1,HSD11B1,G6PC,REN,GPX1,MEST,SMPD1,HDAC2,PLCB1,DCT,SLPI,MMP12,F13A1,ALAS2,DNASE2,DNMT3B,PDHA1,ALDH3A1,SRXN1,PREP,LANCL1,UROD,DDX25,ZC3H12C,ABL2,RFC2,PDE6H,OASL,ATG4C,NVL,DZIP3,GSTM2,NDUFAF1,HHIP,ATP1A2,GALNT11,TMX1,SPATA20,RENBP,INMT,MTMR1,PFKP,ABHD14B,MYO1G,TGM6,SPAG1,POGLUT1,AUH,HARS2	2.67E-08	biological _process	2
**transcription, DNA-dependent**	Group	2265	2265	53	2	ZNF16,ZNF184,ATP1B4,ZNF576,ZNF514,SCRT2,ZNF502,ZNF320,ZNF549,ZNF660,FOXI2,PARP1,KLF9,WDR5,PROX1,HDAC2,HIF3A,GCM1,TBX2,NFIC,ZBTB7A,ZNF350,RLIM,SMYD2,PTF1A,ZNRD1,ZBTB10,AATF,HSF2,MBD1,FOXA3,TFB1M,BRF2,HTATSF1,EGR4,RFXANK,KHDRBS2,DMAP1,BCORL1,SKOR1,VGLL4,DRAP1,BHLHE22,JMJD1C,FIZ1,ZNF395,ARID5A,MTERFD3,ZNF296,MAFIP,MAFK,ZNF140,NCOA5	1.09E-07	biological _process	5
**transport**	Group	1812	1812	46	2	SLC25A44,ATP1B4,ATP5S,SLC48A1,NXT2,SLC45A4,SLC16A13,SLC25A45,LCN12,WASH3P,NXF2B,KCNJ2,SLC20A2,ABCG5,KCNJ11,SLC39A3,TRPA1,SLC28A2,FXN,SLC1A2,AQP7,SERPINA6,KCNA2,SLC5A2,LRP6,RINT1,SLC30A4,FAM38B,SLCO1C1,NUP50,RABIF,UQCRFS1,DDX25,SLC25A30,VPS33B,TRAPPC3,ATG4C,RAB35,FA2H,	8.44E-08	biological _process	4

						RAB33B,ATP1A2,TMX1,NDUFA2,NDUFS7,SLC25A42,THOC3			
**Melanogenesis**	Pathway	50	694	21	3	CALML6,PLCXD2,FGF2,PRKD2,GLP2R,RAPGEF4,VEGFC,LRP5,PLCB1,DCT,LRP6,CCR3,PF4,WNT2,CCL8,ALDH3A1,ADRA2C,DZIP3,WNT8B,INMT,FARS2	0.0349534	Ariadne Cell Signaling Pathways	292
**response to drug**	Group	484	484	20	4	Rad54l,CA9,FOSL1,ABCG5,KCNJ11,REN,TRPA1,MDK,SMPD1,FXN,SLC1A2,HP,AQP7,VEGFC,HDAC2,MMP12,ALAS2,DNMT3B,ALDH3A1,UQCRFS1	4.46E-07	biological_process	6
**transmembrane transport**	Group	831	831	20	2	SLC25A44,SLC45A4,SLC16A13,SLC25A45,SLC20A2,SLC39A3,G6PC,TRPA1,SLC28A2,SLC1A2,AQP7,KCNA2,SLC5A2,SLC30A4,TIMM8A,SLCO1C1,NUP50,SLC25A30,ATP1A2,SLC25A42	0.00087173	biological_process	19
**multicellular organismal development**	Group	1146	1146	20	1	NXF2B,FGF2,LCLAT1,MDK,PROX1,VEGFC,LRP5,LRP6,DNASE2,GCM1,WNT2,TBX2,ZBTB7A,PTF1A,LECT1,FOXA3,TLX2,DDX25,WNT8B,SPATA20	0.026832	biological_process	224
**positive regulation of cell proliferation**	Group	438	438	17	3	NACC2,FGF2,LEP,FOSL1,GLP2R,FXN,PROX1,VEGFC,HDAC2,LRP5,WNT2,CD81,TBX2,TBC1D8,ALDH3A1,EGR4,RPS9	7.50E-06	biological_process	7
**negative regulation of transcription, DNA-dependent**	Group	443	443	16	3	NACC2,FGF2,PROX1,HDAC2,PLCB1,DNMT3B,TBX2,NFIC,ZBTB7A,ZNF350,MBD1,CBFA2T2,DMAP1,SKOR1,BHLHE22,ARID5A	3.32E-05	biological_process	11
**negative regulation of transcription from RNA polymerase II promoter**	Group	506	506	15	2	FOXI2,LEP,PROX1,HDAC2,WNT2,TBX2,NFIC,ZBTB7A,RLIM,SMYD2,ZBTB10,FOXA3,SKOR1,DRAP1,WNT8B	0.00048978	biological_process	15
**positive regulation of transcription from RNA polymerase II promoter**	Group	610	610	15	2	FGF2,PRKD2,TLR3,PROX1,HDAC2,LRP5,LRP6,HIF3A,WNT2,NFIC,AATF,CDON,FOXA3,RFXANK,PPP2R5B	0.00306521	biological_process	40
**proteolysis**	Group	657	657	15	2	CELA2B,LONP2,PRSS42,CARD17,REN,HP,MMP12,ADAM2,TPSG1,PREP,MBTPS2,TRHDE,ATG4C,LCN1,RHBDL2	0.00600808	biological_process	56

**ion transport**	Group	693	693	15	2	ATP1B4,ATP5S,KCNJ2,SLC20A2,KCNJ11,SLC39A3,TRPA1,FXN,SLC1A2,KCNA2,SLC5A2,SLC30A4,FAM38B,SLCO1C1,ATP1A2	0.00954141	biological_process	82
**cell adhesion**	Group	686	686	13	1	PCDHB18,CNTNAP3,CCR3,ADAM2,PTK7,AATF,CDON,COL14A1,ABL2,TPBG,AMTN,CLSTN3,CDH9	0.0388261	biological_process	298
**blood coagulation**	Group	477	477	12	2	PIK3CG,RAPGEF4,SERPINC1,VEGFC,HDAC2,SPARC,F13A1,PF4,SERPINF2,ADRA2C,JMJD1C,MAFK	0.00647078	biological_process	65
**cell cycle**	Group	604	604	12	1	BEX2,CDK6,CCNF,UBE2C,RINT1,CEP250,CDCA5,TXNL4B,SPC24,HAUS6,CDK14,CDKL1	0.0341036	biological_process	289
**ATP catabolic process**	Group	291	291	10	3	DNAH17,ATP1B4,LONP2,TNNT2,ABCG5,RAD51L3,DDX46,DDX25,RFC2,ATP1A2	0.00142783	biological_process	24
**positive regulation of transcription, DNA-dependent**	Group	476	476	10	2	FOXI2,MDK,PROX1,LRP5,PLCB1,LRP6,PTF1A,FOXA3,EGR4,TMX1	0.0368288	biological_process	295
**mitotic cell cycle**	Group	316	316	9	2	CDK6,UBE2C,CEP250,PCNT,CENPJ,RFC2,SPC24,TUBB2C,SDCCAG8	0.00816647	biological_process	73
**lipid metabolic process**	Group	372	372	9	2	PLCXD2,IAH1,LCN12,LEP,ABCG5,HSD11B1,NPC1L1,GPX1,PLCB1	0.0214784	biological_process	162
**spermatogenesis**	Group	423	423	9	2	HP,AQP7,HSF2,FOXA3,DDX25,SBF1,SPATA20,SPATA4,ZNF296	0.0432286	biological_process	359
**Histones Ubiquitylation**	Pathway	23	334	9	2	HIST1H2BG,HDAC2,DNMT3B,UBE2C,MBD1,SAE1,DMAP1,DZIP3,CDY1	0.0142453	Ariadne Cell Process Pathways	140
**TRRAP/Tip60 Chromating Remodeling**	Pathway	35	412	9	2	PARP1,HDAC2,DNMT3B,XRCC4,RAD51L3,MBD1,DMAP1,RFC2,CDY1	0.0481643	Ariadne Cell Process Pathways	370
**transcription from RNA polymerase II promoter**	Group	309	309	8	2	PARP1,FOSL1,PROX1,HIF3A,NFIC,HSF2,MBD1,CSTF1	0.0205827	biological_process	160
**cell division**	Group	336	336	8	2	CDK6,CCNF,UBE2C,CDCA5,CENPJ,SPC24,HAUS6,CDK14	0.0316901	biological_process	235

**G2-M transition of mitotic cell cycle**	Group	116	116	7	6	PLCB1,CEP250,PCNT,CENPJ,CDK14,TUBB2C,SDCCAG8	0.00028789	biological_process	14
**methylation**	Group	144	144	7	4	FTSJD1,SHMT1,DNMT3B,TFB1M,DMAP1,INMT,TRMT2A	0.00104794	biological_process	22
**negative regulation of endopeptidase activity**	Group	166	166	7	4	GPX1,SERPINC1,SERPINA6,SLPI,SERPINF2,SERPINB8,RENBP	0.00236332	biological_process	38
**response to organic cyclic compound**	Group	234	234	7	2	LONP2,FOSL1,GPX1,FXN,HP,ALDH3A1,UROD	0.0146152	biological_process	145
**platelet activation**	Group	242	242	7	2	PIK3CG,CD40LG,VEGFC,SPARC,F13A1,PF4,SERPINF2	0.0172552	biological_process	153
**response to stress**	Group	249	249	7	2	HSPB2,REN,HP,HSF2,MKNK1,TMX1,PPP1R15B	0.0198271	biological_process	157
**mitosis**	Group	252	252	7	2	CCNF,UBE2C,PBK,CDCA5,TXNL4B,SPC24,HAUS6	0.0210073	biological_process	161
**chromatin modification**	Group	262	262	7	2	WDR5,HDAC2,SMYD2,FOXA3,DMAP1,BCORL1,JMJD1C	0.0252926	biological_process	221
**cholesterol metabolic process**	Group	81	81	6	7	LEP,NPC1L1,CYP27A1,LRP5,MBTPS2,CLN6	0.00026017	biological_process	13
**lung development**	Group	121	121	6	4	FGF2,HSD11B1,PROX1,SPARC,WNT2,HHIP	0.00214538	biological_process	35
**female pregnancy**	Group	123	123	6	4	PSG4,LEP,FOSL1,FCGRT,ALAS2,PSG6	0.0023297	biological_process	37
**Wnt receptor signaling pathway**	Group	168	168	6	3	LRP5,LRP6,WNT2,PTK7,WNT8B,CDK14	0.0104505	biological_process	89
**protein ubiquitination**	Group	220	220	6	2	CCNF,UBE2C,RLIM,SAE1,SOCS7,FBXO9	0.0339712	biological_process	288
**response to hypoxia**	Group	241	241	6	2	LEP,CA9,HP,HIF3A,ALAS2,ALDH3A1	0.0491223	biological_process	371
**Double Strand DNA Homologous Repair**	Pathway	30	184	6	3	PARP1,XRCC4,RAD51L3,RBBP8,DMAP1,RFC2	0.0198851	Ariadne Cell Process Pathways	158

**retina morphogenesis in camera-type eye**	Group	10	10	5	50	PROX1,LRP5,LRP6,PTF1A,BHLHE22	3.58E-08	biological _process	3
**cerebral cortex development**	Group	46	46	5	10	MDK,PLCB1,LRP6,BBS2,CDON	0.00014106	biological _process	12
**response to hydrogen peroxide**	Group	68	68	5	7	FOSL1,TRPA1,GPX1,HP,PPP1R15B	0.00088265	biological _process	20
**sodium ion export**	Group	69	69	5	7	ATP1B4,SLC28A2,SLC1A2,SLC5A2,ATP1A2	0.00094324	biological _process	21
**regulation of blood pressure**	Group	75	75	5	6	LEP,REN,LRP5,ATP1A2,RENBP	0.00137381	biological _process	23
**platelet degranulation**	Group	80	80	5	6	VEGFC,SPARC,F13A1,PF4,SERPINF2	0.00183101	biological _process	32
**canonical Wnt receptor signaling pathway**	Group	84	84	5	5	LRP5,LRP6,WNT2,PTK7,WNT8B	0.00226998	biological _process	36
**wound healing**	Group	102	102	5	4	FGF2,DCN,SPARC,F13A1,PTK7	0.00522915	biological _process	53
**regulation of translation**	Group	112	112	5	4	CPEB1,MKNK1,DDX25,RPS9,PPP1R15B	0.00771718	biological _process	71

Table S2.- List of Master Regulator genes identified by Pathway Studio likely to control significant number of genes in sepsis (p> 0.05)

MASTER REGULATORS							
**Name**	**Total # of Neighbors**	**Overlap**	**Percent Overlap**	**Gene Set Seed**	**Overlapping Entities**	**p-value**	**#**
**Expression Targets of FOXN4**	12	4	30	FOXN4	PROX1,TBX2,PTF1A,BHLHE22	5.19E-05	1
**Expression Targets of SPC24**	1	2	100	SPC24	PARP1,SPC24	0.0002889	2
**Expression Targets of SP1**	1251	36	2	SP1	FGF2,DCN,KCNJ2,PARP1,LEP,TNNT2,CES1,CDK6,CA9,FOSL1,KCNJ11,HSD11B1,G6PC,REN,CD40LG,SMPD1,WDR5,SLC1A2,CYP27A1,FCGRT,VEGFC,LRP5,SPARC,ALAS2,DNASE2,SHMT1,DNMT3B,MTA3,TBX2,ZBTB7A,LECT1,KRT17,ALDH3A1,TFB1M,CENPW,ATP1A2	0.001152	3
**Expression Targets of NR1H2**	48	5	10	NR1H2	ABCG5,G6PC,REN,SMPD1,HP	0.00141833	4
**Expression Targets of ANGPTL6**	3	2	50	ANGPTL6	FGF2,G6PC	0.00169463	5
**Expression Targets of endothelin**	101	7	6	endothelin	FGF2,DCN,LEP,FOSL1,REN,SERPINC1,SLC1A2	0.00177199	6
**Expression Targets of CENPJ**	4	2	40	CENPJ	TRAF1,CENPJ	0.0027927	7
**Expression Targets of Mir199b**	4	2	40	Mir199b	REN,Mir199b	0.0027927	8
**Expression Targets of IL1 family**	563	19	3	IL1 family	FGF2,DCN,LEP,FOSL1,HSD11B1,G6PC,REN,CD40LG,CYP27A1,TLR3,HP,VEGFC,TRAF1,SLPI,MMP	0.0035579	9

					12,SERPINF2,CCL8,OSCAR,IL1R APL2		
**Expression Targets of RLIM**	5	2	33	RLIM	HDAC2,RLIM	0.0041421 2	10
**Expression Targets of SUMO2**	5	2	33	SUMO2	HP,SAE1	0.0041421 2	11
**Expression Targets of CDON**	5	2	33	CDON	TNNT2,CDON	0.0041421 2	12
**Expression Targets of FOXA3**	19	3	15	FOXA3	G6PC,SLC28A2,FOXA3	0.0044864 6	13
**Expression Targets of MIR1-1**	188	9	4	MIR1-1	TNNT2,SRXN1,SLC25A30,TRAPP C3,PLEKHG2,PTPLAD1,CDK14,S H2D4A,MTMR12	0.0050835 8	14
**Expression Targets of SP3**	376	14	3	SP3	KCNJ2,PARP1,TNNT2,CA9,KCNJ 11,G6PC,REN,SPARC,DNASE2,D NMT3B,NFIC,LECT1,CENPW,NX NL1	0.0052022 6	15
**Expression Targets of PBX1**	41	4	9	PBX1	FGF2,DCN,REN,PF4	0.0055216 5	16
**Expression Targets of ferritin**	22	3	13	ferritin	HP,ALAS2,SHMT1	0.0067134 5	17
**Expression Targets of VLDLR**	7	2	25	VLDLR	LRP5,LRP6	0.0075600 3	18
**Expression Targets of ABCG8**	7	2	25	ABCG8	ABCG5,NPC1L1	0.0075600 3	19
**Expression Targets of CYP7A1**	25	3	11	CYP7A1	ABCG5,NPC1L1,CYP27A1	0.0094944 6	20
**Expression Targets of EGR3**	25	3	11	EGR3	FGF2,CD40LG,FXN	0.0094944 6	21
**Expression Targets of PBX2**	8	2	22	PBX2	DCN,PF4	0.0096115 9	22
**Expression Targets of HNF1B**	79	5	6	HNF1B	G6PC,SLC28A2,SERPINA6,PTF1	0.0117124	23

					A,VPS33B		
**Expression Targets of MAD2L1**	9	2	20	MAD2L1	PARP1,CDK6	0.0118806	24
**Expression Targets of ABCG5**	9	2	20	ABCG5	ABCG5,NPC1L1	0.0118806	25
**Expression Targets of CES1**	9	2	20	CES1	CES1,CYP27A1	0.0118806	26
**Expression Targets of P2RY11**	9	2	20	P2RY11	LEP,REN	0.0118806	27
**Expression Targets of GALNS**	9	2	20	GALNS	FGF2,DCN	0.0118806	28
**Expression Targets of HNF4A**	216	9	4	HNF4A	ABCG5,KCNJ11,G6PC,NPC1L1,SERPINC1,CYP27A1,SERPINA6,PLCB1,ALDH3A1	0.0120668	29
**Expression Targets of ADRB3**	28	3	10	ADRB3	LEP,G6PC,HP	0.0128551	30
**Expression Targets of GCM1**	10	2	18	GCM1	LEP,GCM1	0.0143592	31
**Expression Targets of FOXM1**	151	7	4	FOXM1	G6PC,CCNF,SERPINA6,MBL2,MMP12,CENPB,FOXA3	0.0152841	32
**Expression Targets of NR1H3**	86	5	5	NR1H3	CES1,ABCG5,HSD11B1,G6PC,REN	0.0163747	33
**Expression Targets of IAPP**	31	3	9	IAPP	PARP1,LEP,REN	0.016813	34
**Expression Targets of ONECUT1**	32	3	9	ONECUT1	G6PC,SERPINA6,PTF1A	0.0182669	35
**Expression Targets of ANG**	12	2	15	ANG	FGF2,VEGFC	0.0199143	36
**Expression Targets of TDGF1**	13	2	14	TDGF1	FGF2,PARP1	0.0229759	37
**Expression Targets of MC2R**	13	2	14	MC2R	LEP,G6PC	0.0229759	38
**Expression Targets of AQP4**	13	2	14	AQP4	SLC1A2,AQP7	0.0229759	39
**Expression Targets of PPARG**	588	17	2	PPARG	FGF2,DCN,LEP,HSD11B1,G6PC,REN,GPX1,MEST,CD40LG,FXN,SLC1A2,CYP27A1,HP,AQP7,MMP12,CCL8,ALDH3A1	0.0235477	40
**Expression Targets of USF1**	165	7	4	USF1	DCN,G6PC,REN,PF4,OSCAR,TBX2,HSF2	0.0235488	41
**Expression Targets of ASCL1**	64	4	6	ASCL1	PROX1,DCT,PTF1A,BHLHE22	0.0247109	42

**Expression Targets of MITF**	97	5	5	MITF	DCT,TPSG1,OSCAR,TBX2,IL18R1	0.0259482	43
**Expression Targets of PCK2**	14	2	13	PCK2	HSD11B1,G6PC	0.0262174	44
**Expression Targets of LRP5**	14	2	13	LRP5	LRP5,LRP6	0.0262174	45
**Expression Targets of LECT1**	14	2	13	LECT1	CDK6,LECT1	0.0262174	46
**Expression Targets of NR3C1**	373	12	3	NR3C1	FGF2,DCN,HSPB2,HSD11B1,G6PC,KLF9,MDK,CD40LG,SLC1A2,HP,SLPI,AKAP12	0.0265482	47
**Expression Targets of endopeptidase inhibitor**	98	5	5	endopeptidase inhibitor	LEP,HSD11B1,CCKAR,CD40LG,CD3E	0.0269636	48
**Expression Targets of HNF1A**	211	8	3	HNF1A	G6PC,NPC1L1,SLC28A2,CYP27A1,SERPINA6,SLC5A2,CD3E,ALDH3A1	0.0288044	49
**Expression Targets of FABP**	15	2	12	FABP	LEP,TNNT2	0.0296317	50
**Expression Targets of acute-phase protein**	15	2	12	acute-phase protein	SERPINC1,HP	0.0296317	51
**Expression Targets of PTGES**	15	2	12	PTGES	G6PC,REN	0.0296317	52
**Expression Targets of INPPL1**	15	2	12	INPPL1	PARP1,G6PC	0.0296317	53
**Expression Targets of ARNT**	68	4	5	ARNT	LEP,CA9,G6PC,ALDH3A1	0.0299554	54
**Expression Targets of PTGER1**	39	3	7	PTGER1	FGF2,CD40LG,VEGFC	0.0303441	55
**Expression Targets of HSF2**	16	2	11	HSF2	HSPB2,HSF2	0.0332121	56
**Expression Targets of PTF1A**	16	2	11	PTF1A	PROX1,PTF1A	0.0332121	57
**Expression Targets of gap-junction protein**	16	2	11	gap-junction protein	REN,SLC1A2	0.0332121	58
**Expression Targets of RXRA**	178	7	3	RXRA	FGF2,ABCG5,HSD11B1,NPC1L1,GPX1,SERPINC1,AQP7	0.0336021	59
**Expression Targets of IL1B**	1001	25	2	IL1B	FGF2,DCN,CA9,FOSL1,HSD11B1,REN,MDK,GPX1,WDR5,CYP27A1,TLR3,HP,FCGRT,VEGFC,TRAF1,SLC5A2,SPARC,SLPI,MMP12,F13	0.0342653	60

					A1,AKAP12,CCR3,SOCS7,GPR78,IL18R1		
**Expression Targets of KRT14**	17	2	11	KRT14	VEGFC,KRT15	0.0369521	61
**Expression Targets of LEPR**	43	3	6	LEPR	LEP,HSD11B1,G6PC	0.0387284	62
**Expression Targets of oncogene**	312	10	3	oncogene	FGF2,DCN,LEP,FOSL1,VEGFC,SPARC,AKAP12,DNMT3B,CCL8,RBBP8	0.0419128	63
**Expression Targets of PTBP1**	45	3	6	PTBP1	FGF2,CD40LG,TRAF1	0.043317	64
**Expression Targets of IL1A**	358	11	3	IL1A	FGF2,DCN,HSPB2,LEP,SLC20A2,HSD11B1,VEGFC,TRAF1,DCT,SLPI,KRT17	0.0435825	65
**Expression Targets of MYF6**	19	2	10	MYF6	TNNT2,TNNT1	0.0448849	66
**Expression Targets of TCF4**	19	2	10	TCF4	LEP,DCT	0.0448849	67
**Expression Targets of IGF1**	592	16	2	IGF1	PARP1,LEP,CDK6,FOSL1,SLC20A2,HSD11B1,REN,KLF9,GLP2R,MDK,CYP27A1,SERPINA6,VEGFC,SPARC,SLPI,PBK	0.0464964	68
**Expression Targets of MMP13**	20	2	9	MMP13	DCN,WDR5	0.0490655	69
**Expression Targets of estrogen receptor**	321	10	3	estrogen receptor	FGF2,HSPB2,LEP,TNNT2,CDK6,NPC1L1,CD40LG,SLC1A2,MTA3,TNNT1	0.0491099	70
